# In vitro measurements of ultrafiltration precision in hemofiltration and hemodialysis devices used in infants

**DOI:** 10.1007/s00467-022-05439-y

**Published:** 2022-03-29

**Authors:** Jean Crosier, Mike Whitaker, Heather J. Lambert, Paul Wellman, Andrew Nyman, Malcolm G. Coulthard

**Affiliations:** 1 Great North Children’s Hospital, Queen Victoria Road, Newcastle upon Tyne, NE1 4LP UK; 2 Northern Medical Physics and Clinical Engineering, Royal Victoria Infirmary, Newcastle upon Tyne, NE1 4LP UK; 3 Evelina London Children’s Hospital, Westminster Bridge Road, London, SE1 7EH UK

**Keywords:** Infant, Hemodialysis, Hemofiltration, Ultrafiltration, Precision, Prismaflex, Aquarius, NIDUS, CARPEDIEM, Aquadex

## Abstract

**Background:**

To determine in vitro whether infant hemofiltration and hemodialysis devices can reliably deliver precise ultrafiltration (UF) control.

**Methods:**

We tested the Prismaflex, Aquarius and NIDUS devices which have different circuit types, by in vitro testing with a bag of saline set up as a dummy patient, and monitoring fluid shifts by precise weighing. We looked for differences between the UF rates set and achieved and between the UF result the device displays to the clinician and the true volumes removed, which may lead to clinical errors. We performed short studies at UF settings of zero and 40 ml/h, and with and without simulating poor withdrawal and return lines, and simulated a 4-h treatment session.

**Results:**

The Prismaflex setting vs actual errors and display vs actual errors had wide variances, with SDs of 4.1 and 14.0 ml by 15 min, respectively, at both zero and 40 ml/h UF settings. The Aquarius values were wider at 17.3 and 30.3 ml, respectively. For the NIDUS, the mean UF errors were close to zero, and the variances were 0.17 ml. Stop-alarms induced by an obstructed line produced extra UF errors of up to 0.2 ml. A limitation was that we used crystalloid and not colloid for these tests.

**Conclusions:**

Hemotherapy devices with conventional circuits available in the UK do not regulate UF control sufficiently well to recommend for use in small infants, but the NIDUS volumetrically controlled circuit does. All hemotherapy devices intended for small infants should be tested for UF precision. We were unable to test the CARPEDIEM or Aquadex devices.

**Graphical Abstract:**

A higher resolution version of the Graphical abstract is available as Supplementary information

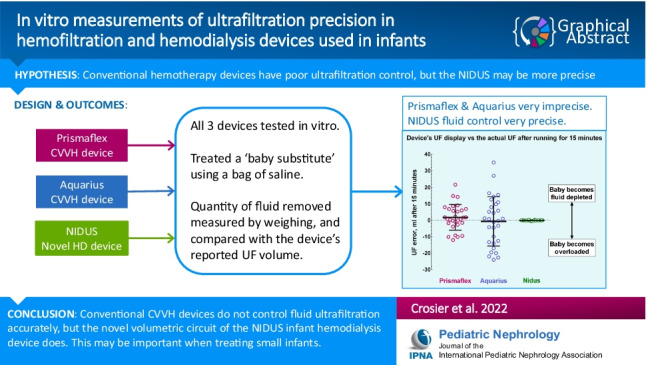

**Supplementary Information:**

The online version contains supplementary material available at 10.1007/s00467-022-05439-y.

## Introduction

Hemotherapy (HT) machines (dialysis and filtration devices) must continuously regulate fluid removal (ultrafilter; UF) from patients because fluid accumulation can cause hypertension and pulmonary edema, while rapid withdrawal can lead to hypovolemic shock. Concern has been expressed that conventional HT device fluid balance control systems limit their capacity to either measure or adjust precisely how much fluid they UF [1], making them potentially unsafe for use in infants. As a consequence, HT devices had their licenses revoked for use in children weighing < 20 kg in the USA (< 8 kg in Europe) in 2006, when the US Food and Drug Administration (FDA) reported that the UF errors of pediatric Prisma devices of ± 60 ml/h had led to at least nine deaths and eleven serious injuries [2]. The manufacturers of the upgraded Prismaflex currently claim a UF imprecision of ± 30 ml/h [3], and they remain unlicensed for infant use. However, the lack of alternative devices has meant that infants continue to be treated off-license with conventional HT machines across the world. We have previously demonstrated that even relatively stable infants receiving standard intermittent hemodialysis may suffer symptomatic hypovolemia due to large, unpredictable UF errors [4].

Currently in the UK, most babies weighing < 8 kg who undergo HT are treated off-license with continuous veno-venous hemofiltration (CVVH) using either the Prismaflex or the Aquarius devices, or the novel Newcastle infant hemodialysis and ultrafiltration system (NIDUS) for those in the IKID (Infant Kidney Dialysis and filtration) study. The IKID study is funded by the Efficiency and Mechanism Evaluation programme of the National Institute for Health Research, is designed to compare current practice including peritoneal dialysis with NIDUS [5] and has completed recruitment and will be reporting shortly. The NIDUS has been designed to treat babies of between 800 g and 8 kg and uses a volumetrically controlled syringe-driven circuit to regulate fluid balance, rather than using circuit pressures to regulate the UF control algorithms as occurs in conventional HT devices. The precision of UF control will be the primary outcome measure for the IKID study, measured by the gain in the combined weight of the bags containing dialysis, replacement and waste fluids.

In this study, we have undertaken in vitro comparisons of the precision of UF control of the Prismaflex, Aquarius and NIDUS devices by setting them each to ‘treat’ a bag of saline instead of a baby and continuously weighing it to measure the true fluid shifts that they generate. We consider separately two types of imprecision errors. The first is the difference between the UF rate set by the user and the rate actually achieved by the device (setting vs. actual UF error), and the second is the difference between the UF volume that the device’s display reports it has achieved, and the actual volume removed (display vs. actual UF error). Both errors have important implications, but the second one is likely to be especially misleading because most clinicians will assume that the device’s displayed UF volume is accurate, and will base treatment decisions on that information.

## Method

### Experimental setup

We primed each HT device with normal saline using a pediatric circuit and filter and operated them as we normally would to deliver either CVVH (the Prismaflex and Aquarius) or dialysis (the NIDUS) to a baby of about 4 kg, apart from not using any anticoagulants (Table 1). However, instead of connecting their circuits to a baby we attached them to a bag of saline suspended from a weigh-scale at the height that a baby would normally be nursed. The scales were highly stable and precise to ± 0.1 g and allowed us to monitor the fluid volume shifts due to ultrafiltration, assuming the density of normal saline to be exactly 1 g/ml (which introduces a 0.5% error). During the study periods, we set the devices to deliver UF rates of either zero or 40 ml/h.

**Table 1:** Hemotherapy machine filter details and settings used during the in vitro studies

Machine settings	Prismaflex	Aquarius	NIDUS
Filter	HF20	HF03	NeoFlux1
Filter surface area (m^2^)	0.2	0.3	0.045
Total circuit volume (ml)	60	96	14.8
Blood flow (ml/min)	50	50	20
Fluid replacement (ml/h)	150	150	-
Dialysate flow (ml/h)	-	-	400

### Study periods

First, we studied the machines during a series of 15 min test runs, in three conditions: with no added resistance to the access lines, or with resistance added either to the sampling line or the return line to simulate different clinical scenarios. These were achieved by clamping the access tubing sufficiently to generate line pressures of between ± 150 and ± 300 mmHg. The devices were tested ten times in each condition, five with them set not to remove any fluid, and five set to remove an UF of 40 ml/h. Second, we set each device to deliver a four-hour treatment, with no UF for the first half, and a rate of 40 ml/h for the second two hours, with recordings taken approximately every 5 min. For the NIDUS, the recordings were taken at the same point in each of the approximately two-minute cycles. The Prismaflex and Aquarius were tested in Newcastle and the Evelina Hospital, respectively. The preliminary short test runs on the NIDUS were completed in Newcastle, but both centers carried out a four-hour NIDUS study.

### Measurements

At each time point we recorded the UF rate set, the device’s display of the UF volume achieved, and the UF recorded by weighing the saline bag, allowing us to calculate both the setting vs. actual UF errors and the display vs. actual UF errors. Both types of error are reported in relation to the devices, thus as being positive UF when the ‘baby’ lost more fluid than was set or displayed (the clinical equivalent of risking fluid depletion), and as being negative when they were delivered extra unrecognized fluid (risking overload).

### Statistics

In the 15-minute studies, we used unpaired t-tests to compare different data sets, or to determine whether the data set means differed from zero, and we used the F test to compare their variances. We plotted these data with means and 1 standard deviation (SD) error bars. In the four-hour studies, we have simply plotted the results for visual comparison.

## Results

### The 15-min studies

**Fig. 1:**
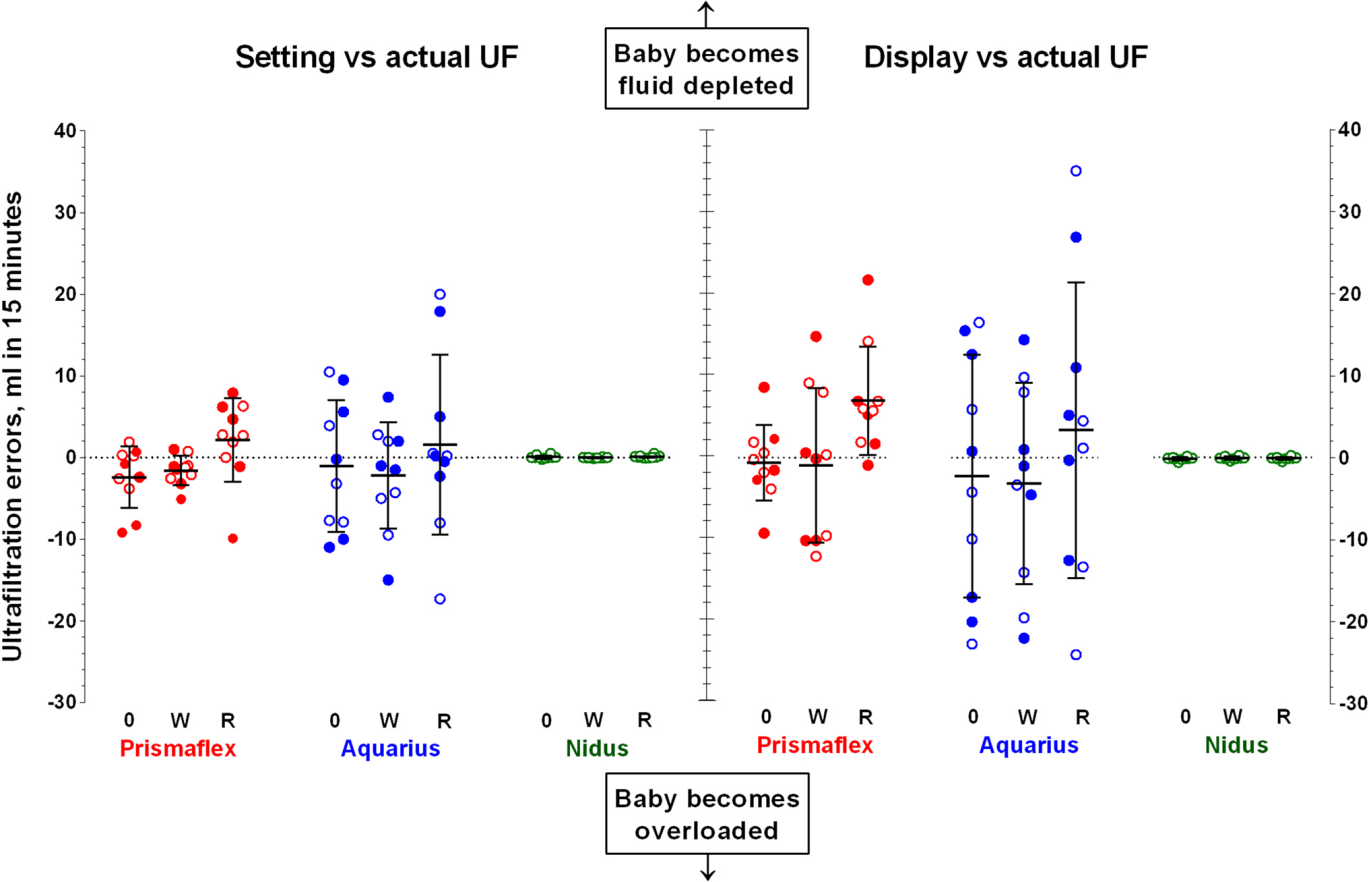
In vitro measurements of ultrafiltration (UF) imprecision produced by three hemotherapy devices, measured over 15-min intervals, and expressed as ml per 15 min. The left panel shows the setting vs. actual UF achieved errors, and the right panel shows the display vs. actual UF errors. For the Prismaflex and Aquarius data, the open symbols indicate when the UF rate was set at zero, and the closed ones were at 40 ml/h, but the symbols were too closely bunched to allow this differentiation to be shown for the NIDUS. The columns indicate studies where either no resistance was applied to the access lines (0), or where it was applied to the withdrawal (W) or the return (R) line. The error bars indicate mean ± 1SD

The setting vs. actual and display vs. actual errors measured during the ¼-hour study periods for each device are shown in Fig. 1. Note that the error bars indicate ± 1 SD and that there are separate plots for when a resistance was applied to the withdrawal (W) or return (R) access lines, or to neither (0), and where the open and filled circles represent UF rates of 0 and 40 ml/h, respectively (this could not be applied to the NIDUS data as the symbols were too crowded). The mean values for UF errors ranged around zero for the Prismaflex and Aquarius, apart from when the Prismaflex met a return line resistance when the device generated excessive unrecorded fluid losses (t, *p* = 0.04 for setting vs. actual errors, *p* = 0.01 for display vs. actual errors). The NIDUS UF errors had mean values and 95% confidence intervals (CI) close at zero in all conditions, at 0.05 (–0.05 to 0.15) ml for setting vs. actual errors, and –0.05 (–0.15 to 0.05) ml for display vs. actual errors. The variance of the NIDUS results was low, with SDs of 0.17 ml for both types of error. The variance of the Prismaflex errors was significantly higher (F, *p* < 0.0001) with an SD of 4.1 ml for the setting vs. actual UF errors and higher still (F, *p* = 0.002) at an SD of 14.0 ml for its display vs. actual UF achieved errors. Note that this indicates that 95% of Prismaflex readings are likely to differ from the true clinical situation by up to ± 28 ml within a quarter of an hour of therapy, which is close to the ± 30 ml error that the manufacturer’s claim may occur over one hour. The variances of the Aquarius device were approximately twice as high as for the Prismaflex (F, *p* = 0.01), with setting vs. actual and display vs. actual error SDs of 17.3 and 30.3 ml. In all three devices, the means and variances were unaffected by whether they were programmed to maintain a neutral fluid balance or to remove 40 ml/h.

Increasing the resistance of the withdrawal lines did not influence the means or variances of the Prismaflex or Aquarius fluid balance errors, but a higher return line resistance resulted in both types of error becoming more positive (causing more UF from the ‘baby’) with the Prismaflex (t, *p* = 0.02). The same trend was seen for the Aquarius, but the change was not statistically significant, possibly because of the greater variation in the results. Increasing the resistance of the NIDUS access line did not alter its fluid handling precision unless the flow restrictions were so severe that they triggered an alarm-stop state by exceeding a pressure of ± 400 mmHg. Under these conditions, the compliance of the circuit tubing generated systematic UF errors. Low-pressure blood withdrawal alarms leave a segment of partially collapsed tubing which must be dissipated by releasing a pinch-valve, and that allowed 0.20 ml (CI, 0.06 to 0.34; t-test versus 0, *p* = 0.02) of dialysis fluid to flow across the filter into the circuit. The NIDUS will not allow dialysis to restart until this is done, but fails to record the fluid shift, so produces a –0.20 ml UF error. The opposite happens with high-pressure blood return alarms, when a 0.08 ml UF error occurs (CI, 0.07 to 0.09; t-test, *p* = 0.0001).

### The four-hour sessions

**Fig. 2:**
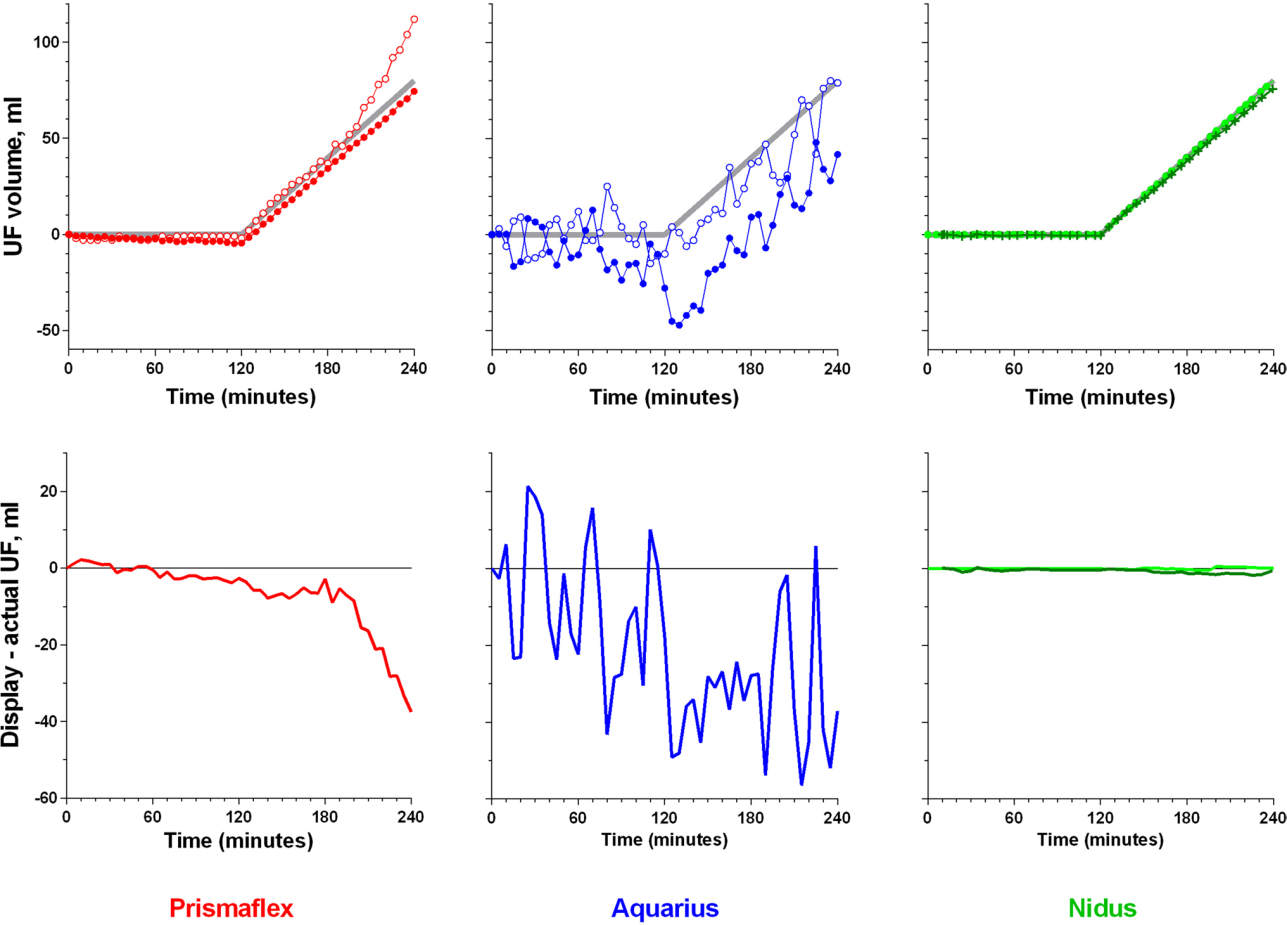
In vitro measurements of ultrafiltration (UF) delivered by three hemotherapy devices over 4 h, set to maintain fluid neutrality for the first 2 h, and then to UF at 40 ml/h. The top three graphs show the UF rate set (grey lines), the UF recorded by the device (filled circles) and the UF actually delivered (open circles), except for the NIDUS plot in which the Newcastle data are shown as green circles, and the Evelina data as dark green crosses. The lower three graphs show the display vs. actual UF errors. The NIDUS results are color-coded as for the top graphs

The upper graphs in Fig. 2 show the UF rates that were set and reported by the three devices, and the UF volumes actually achieved during the four hour ‘treatment’ sessions. It is clear that for the Prismaflex and the Aquarius, the types of errors that were identified during the quarter-hour sessions occurred throughout the whole four hours, in an apparently random way, while the NIDUS remained extremely stable. The lower graphs in Fig. 2 show the display vs. actual errors during the sessions and emphasize the size of the difference between the information presented to the clinician and the true fluid balance; the ‘baby’ on the Prismaflex became 37.5 ml overloaded, the ‘baby’ on the Aquarius was exposed to repeated swings of up to ± 60 ml in either direction, while the NIDUS ‘baby’ developed a maximum discrepancy of just 2.6 ml.

## Discussion

This in vitro study confirms that the Prismaflex and the Aquarius cannot deliver reliable fluid balance even when they are set to produce zero UF. These two HT devices are commonly used off-license in the UK to treat babies < 8 kg. The greatest concern is the size and erratic variations in the discrepancies between the UF volumes that the devices display, and the fluid shifts that have actually occurred. The size of these precision errors are large in comparison with an infant’s total blood volume and thus have the potential to be clinically significant. Indeed, the FDA withdrawal of license for the original PRISMA device use in children under 20 kg was related to poor volume control and was implicated in contributing to several deaths fifteen years ago [2]. The technical challenges of regulating the UF rates of HT devices with conventional circuits are huge [1]; it is hard to precisely measure the addition of small fluid volumes to dialysate or replacement fluids and difficult for computer algorithms to predict the exact trans-membrane pressure that will reliably generate an exact filtration volume. By contrast, the in vitro testing of the NIDUS volumetric UF control system showed only trivial differences between the reported and achieved UF volumes. The only situation where this would not be the case is if frequent stop-alarms were triggered due to obstruction during blood withdrawal or return, as these introduce systematic errors of -0.2 or 0.08 ml, respectively. However, these small errors are seldom seen clinically because the NIDUS instantaneously slows its blood withdrawal and return rates if it is connected to a high-resistance access line, which prevents stop-alarms from occurring except when the line becomes fully obstructed [4].

It is not possible to know the fluid balance imprecision of HT devices without specifically measuring it, so many clinical users may remain unaware of the size of the error that these devices may have. In addition, the devices are frequently used in unwell and clinically unstable children, so observed changes in clinical parameters may have multiple causes. The likely performance of conventional HT machines in the complex task of fluid removal cannot be estimated from the known precision of individual device components, such as the weigh-scales or pumps. UF control can be tested for gravimetrically in three different ways. First, the patient can be weighed before and after a dialysis treatment; we have reported that the NIDUS was very precise during 4-h clinical dialysis sessions in 6 kg babies, while a conventional Gambro AK200 pediatric dialyzer caused large fluid shifts that produced hypotension which needed urgent therapy [4]. A second approach is to weigh the dialysate, replacement and waste fluid bags before and after a dialysis session to determine the quantity of fluid added due to UF from the baby—this is the technique used in the IKID study [5]. The third, and simplest, approach is to do an in vitro study of the change in weight of a saline bag during ‘treatment’ as we have done here. To our knowledge, the UF precision of pediatric hemotherapy devices is not routinely appraised as part of the CE marking or FDA approval processes.

An important shortcoming of this study is that we were unable to evaluate the Aquadex device which has been used off-license in the USA [6] or the CARPEDIEM which is CE marked and available on mainland Europe [7], but which Medtronic did not give permission for us to test. The CARPEDIEM has a conventional circuit that has been miniaturized to provide CVVH in infants, which they report has weigh-scales accurate to 1 g, but fluid replacement or dialysate pump flow errors of ± 7.5% (equivalent to UF errors of ± 10 ml/h at the settings we used), and to have ‘ultrafiltration accuracy … within 1 g/h’. However, it is not clear exactly how this was evaluated, nor whether they are referring to program-type errors, or to clinical-type errors which cannot be detected without specific testing, as described above. We did not test the proposed Japanese ultra-small circuit [8] because it has no method of regulating UF [9].

An additional shortcoming of this study is that it was performed in vitro on saline and does not imply that any of these devices would always be able to ultrafilter 40 ml/h of fluid from any particular baby in all clinical settings. Similarly, our variations of the circuit resistance using saline may not have accurately represented the pressure change fluctuations that occur in vivo. The NIDUS control system has been programmed to limit its UF rate under certain operating pressure conditions, but displays its computer-readjusted UF rate to the operator if these conditions are met.

## Conclusion

This in vitro study confirmed that the Prismaflex and the Aquarius machines, which are the two most widely used CVVH devices for treating babies off-license in the UK, do not accurately regulate UF or correctly inform the clinician of the true fluid balance errors that they generate. The volumetrically controlled NIDUS has been shown in two UK centers to have negligible fluid balance errors in vitro. We have not been able to test the Aquadex or the CARPEDIEM. All HT devices designed to be used in infants should be tested in vitro for their precision of UF control.

## Supplementary Information

Below is the link to the electronic supplementary material.

Graphical abstract (PPTX 157 KB)
