# Correction to: In vitro measurements of ultrafiltration precision in hemofiltration and hemodialysis devices used in infants

**DOI:** 10.1007/s00467-022-05623-0

**Published:** 2022-06-08

**Authors:** Jean Crosier, Mike Whitaker, Heather J. Lambert, Paul Wellman, Andrew Nyman, Malcolm G. Coulthard

**Affiliations:** 1 Great North Children’s Hospital, Queen Victoria Road, Newcastle upon Tyne, NE1 4LP UK; 2 Northern Medical Physics and Clinical Engineering, Royal Victoria Infirmary, Newcastle upon Tyne, NE1 4LP UK; 3 Evelina London Children’s Hospital, Westminster Bridge Road, London, SE1 7EH UK

**Correction to: Pediatr Nephrol**

**https://doi.org/10.1007/s00467-022-05439-y**

The authors would like to make an amendment to their conflict of interest declaration to specify that author Heather J. Lambert has not the potential to receive royalty payments and thus, has no conflict of interest to declare. The corrected conflict of interest declaration is:

“JC and MGC have the potential to receive royalty payments from the sale of NIDUS, and that HJL, MW, PW and AN have no conflict of interest to declare.”

The original article has been corrected.

