# Supplementary material for: In vitro measurements of ultrafiltration precision in hemofiltration and hemodialysis devices used in infants
Source: Pediatr Nephrol. 2022 Mar 29;37(12):3189–94. doi: 10.1007/s00467-022-05439-y (PMC9587064; doi:10.1007/s00467-022-05439-y)
Supplement: Supplementary file 1 — (PPTX 157 KB) [file 467_2022_5439_MOESM1_ESM.pptx]

## Slide 1
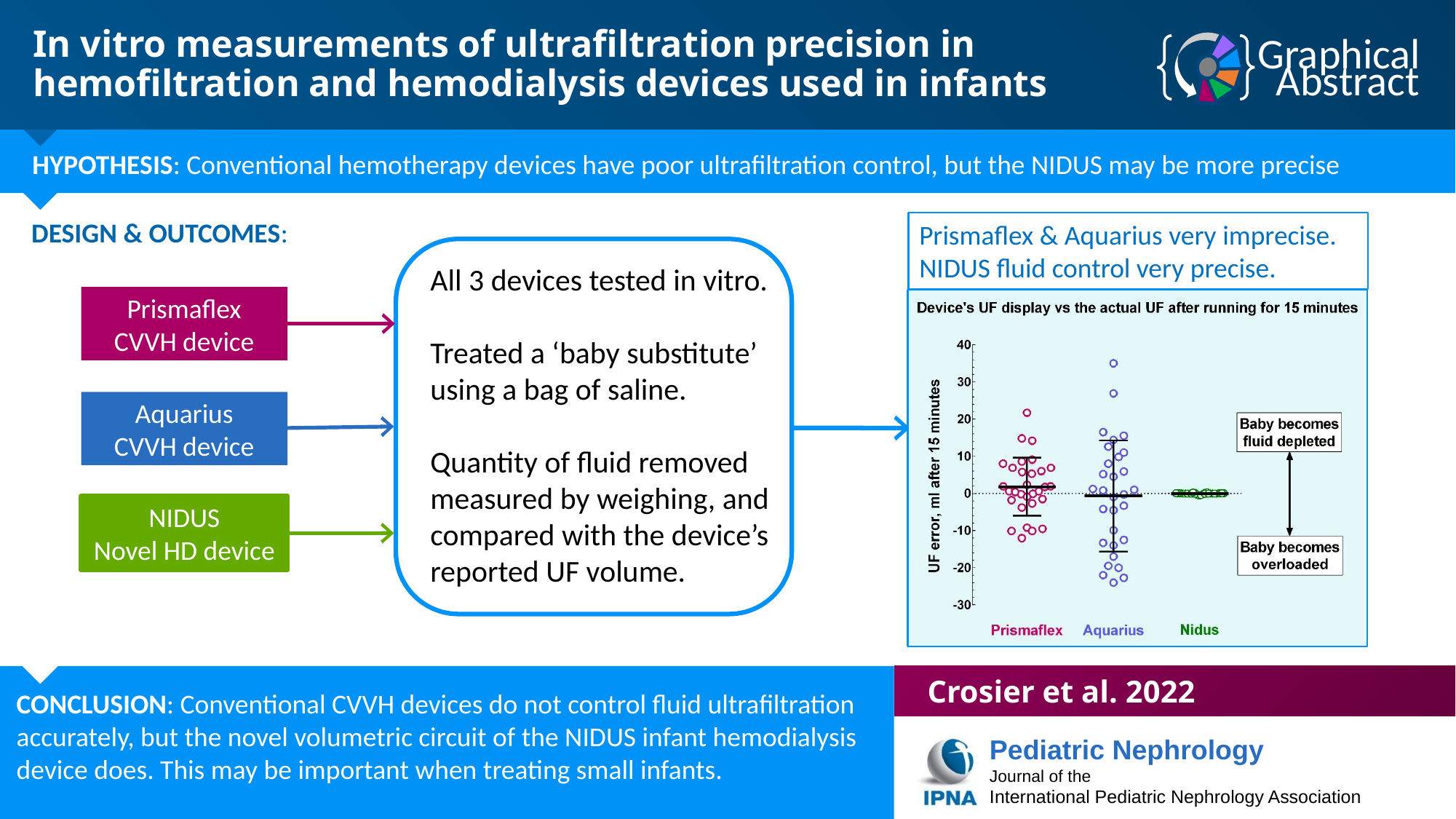

In vitro measurements of ultrafiltration precision in hemofiltration and hemodialysis devices used in infants
HYPOTHESIS: Conventional hemotherapy devices have poor ultrafiltration control, but the NIDUS may be more precise
DESIGN & OUTCOMES:
Prismaflex & Aquarius very imprecise.
NIDUS fluid control very precise.
All 3 devices tested in vitro.
Treated a ‘baby substitute’
using a bag of saline.
Quantity of fluid removed
measured by weighing, and
compared with the device’s
reported UF volume.
Prismaflex
CVVH device
Aquarius
CVVH device
NIDUS
Novel HD device
Crosier et al. 2022
CONCLUSION: Conventional CVVH devices do not control fluid ultrafiltration accurately, but the novel volumetric circuit of the NIDUS infant hemodialysis device does. This may be important when treating small infants.
